# The efficacy and safety of platelet rich plasma and hyaluronic acid in the treatment of knee osteoarthritis: a meta-analysis

**DOI:** 10.3389/fsurg.2026.1725534

**Published:** 2026-03-11

**Authors:** Feimeng An, Huiqiang Wu, Junqian Wang, Genli Zhang, Kai Yan, Feng Wu, Jianzhong Wang, Haibin Zhang

**Affiliations:** 1 Department of Rehabilitation Medicine, Inner Mongolia Hospital of Traditional Chinese Medicine, Hohhot, Inner Mongolia, China; 2 Department of Orthopedics, The Second Affiliated Hospital of Inner Mongolia Medical University, Hohhot, Inner Mongolia, China

**Keywords:** hyaluronic acid, knee osteoarthritis, intra-articular injection, meta-analysis, platelet rich plasma

## Abstract

**Purpose:**

To investigate the efficacy and safety of intra-articular injection ratio of platelet rich plasma (PRP) and hyaluronic acid (HA), and provide evidence-based strategies for the treatment of knee osteoarthritis (KOA).

**Methods:**

Search PubMed, Web of Science, China National Knowledge Infrastructure (CNKI), and Wanfang databases to retrieve literature published from the beginning of database establishment to October 2024. Include in published randomized controlled trials (RCTs) or cohort studies. The research subjects are KOA patients, with the experimental group receiving intra-articular injection of PRP and the control group receiving intra-articular injection of HA. Quality evaluation of the inclusion of Cochrane Handbook risk assessment tools using RevMan5 Perform meta-analysis on outcome measures using three software.

**Results:**

18 articles were included, with a total of 1,326 patients. PRP showed significantly better WOMAC scores at 6 months (SMD=−8.32, *P* < 0.0001) and 12 months (SMD=−3.15, *P* < 0.0001), superior IKDC scores at 6 months (SMD=0.85, *P* = 0.004), and greater pain reduction on VAS at 3 months (SMD=−0.56, *P* < 0.0001) and 6 months (SMD=−0.85, *P* < 0.0001). EQ-VAS scores also favored PRP at 2 months (SMD=0.20, *P* = 0.04) and 12 months (SMD=0.35, *P* = 0.001). No significant differences were found in adverse events (OR = 1.31, *P* = 0.21) or patient satisfaction (MD = 1.60, *P* = 0.08), indicating comparable safety profiles.

**Conclusion:**

PRP has a good clinical therapeutic effect on KOA. Based on this meta-analysis, compared with simple intra-articular injection of HA, PRP can improve WOMAC score, VAS score, and IKDC index score after 6 months of treatment, and enhance patients’ health status. In terms of the incidence of adverse events, the safety of the two treatment options is similar.

## 1 Introduction

As the most common and prone site of osteoarthritis, the knee joint is estimated to have 642 million people suffering from knee osteoarthritis by 2050 (1–3). At present, the mainstream treatment approach is to perform tiered treatment based on the severity of the disease, and there is no drug therapy that can stop or reverse the progression of OA (4). Conservative treatment plans usually only provide temporary relief of pain symptoms, but may have harmful local and systemic consequences, and may not be ideal for patients with severe conditions (5, 6).

Osteoarthritis was once considered a cartilage degeneration disease caused solely by mechanical factors, but with further research, it has gradually been discovered that the disease is caused by a combination of complex risk factors, ultimately affecting the anatomy and function of the entire joint (7, 8). Among them, the structure and biochemical composition of articular cartilage are strictly regulated by chondrocytes, and with changes in the chemical and mechanical environment, chondrocytes also regulate accordingly. Once chondrocytes are activated, many related inflammatory factors will be released, including cytokines such as IL-lp, IL-6, TNF-a, and matrix degrading enzyme ADAMTS (9, 10). As osteoarthritis progresses, subchondral bone also undergoes related changes, such as bone spurs and subchondral bone cysts, and endochondral ossification occurs as blood vessels grow into the corresponding cartilage. Subchondral bone is covered with abundant neural tissue, so as arthritis progresses, patients often experience pain symptoms (11–13).

Platelet rich plasma (PRP) contains various growth factors, including vascular endothelial growth factor (VEGF), epidermal growth factor (EGF), transforming growth factor (TGF) - *β*, platelet-derived growth factor (PDGF), etc (14). In basic research, the potential effectiveness and good biocompatibility of PRP in cartilage repair have been confirmed by many studies (15). PRP refers to obtaining human peripheral blood and centrifuging it to obtain plasma components rich in platelets. When used, thrombin is added to turn it into jelly, so it is also called platelet rich gel (PLG) (16–18). Basic research has shown that the mechanism of PRP treatment for knee osteoarthritis can be summarized as follows: ① promoting extracellular matrix synthesis, providing a suitable microenvironment for chondrocyte proliferation, and promoting massive chondrocyte proliferation; ② inhibiting the expression of local inflammatory factors in the body, reducing local inflammatory reactions, improving joint function, and alleviating joint pain symptoms; ③ forming a three-dimensional environment after activation, which is conducive to the aggregation and action of relevant repair factors, and helps with joint cartilage repair. Hyaluronic acid (HA) hydrogel has been routinely used in clinical practice due to its high hydration, good biocompatibility, chemical and physical properties and ability to simulate the extracellular matrix (ECM) environment (19, 20). Hyaluronic acid, as a type of mucopolysaccharide, is widely present in connective tissue and joint fluid. It can aggregate proteoglycans on the surface of joint cartilage and cells, effectively increasing the viscoelasticity of joint fluid, and has analgesic and anti-inflammatory effects (21, 22).

Although the mainstream view currently holds that PRP treatment for knee osteoarthritis has numerous advantages over traditional treatment, there are still a few scholars who have conducted similar experiments and come to different conclusions, even believing that PRP treatment has no advantages or is even worse than traditional treatment (23, 24). There are still a small number of scholars who have doubts about the efficacy of PRP, and even come to contradictory conclusions through experiments. Filardo et al (25). Collected 192 patients with knee osteoarthritis and randomly assigned them to receive PRP or HA treatment. Patients were followed up before and after injection, but it was found that both groups of patients showed improvement in knee joint function and pain symptoms after treatment, and there was no statistical difference.

Systematic review/Meta-analysis can reduce bias caused by random errors in individual studies, comprehensively display all evidence, and is recognized as the highest level of evidence for evaluating clinical efficacy. It is the literature basis for forming clinical decision-making and practice guidelines (26). The purpose of this study is to compare the efficacy and safety of PRP and HA in adult knee osteoarthritis patients through meta-analysis, and to explore the most effective and safe use regimen.

## 2 Data and methods

### 2.1 Literature search

Plate rich plasma, Key words include “knee osteo, arthritis, hyaluronic acid, PRP, KOA, HA”. Search PubMed database, Embase, Cochrane, China Biology Medicine disc (CBM), China National Knowledge Infrastructure (CNKI) full-text database, Wanfang database, VIP Chinese Science and Technology Periodical Database(VIP) and retrieve literature published from the initial establishment of the database to October 2024.

### 2.2 Inclusion and exclusion criteria

#### 2.2.1 Inclusion criteria

① Research type: Systematic review/Meta-analysis of platelet rich plasma and hyaluronic acid in the treatment of knee osteoarthritis based on randomized controlled trials published in domestic and foreign journals, language limited to Chinese and English; ② Research subjects: Knee osteoarthritis patients with clear diagnostic criteria, without limitations on baseline factors such as region, race, age, gender, and Kellgren Lawrence grading; ③ Intervention measures: The experimental group only received intraluminal injection of platelet rich plasma, while the control group only received injection of hyaluronic acid, without limiting factors such as dosage, model, preparation method, injection interval, etc;

#### 2.2.2 Exclusion criteria

① Duplicate publications; ② Incomplete data; ③ Design scheme for system evaluation/Meta-analysis; ④ Network meta-analysis; ⑤ Narrative review; ⑥ Non final publication journal.

### 2.3 Outcome measures

Western Ontario and McMaster University OA Index Score (WOMAC); International Knee Documentation Committee Knee Assessment Form (IKDC); Visual Analog Scores (27); Adverse reactions of the Health Index Scale (EQ-VAS).

### 2.4 Literature screening and data extraction

Two evaluators independently conduct literature screening, data extraction, and cross checking based on inclusion and exclusion criteria. In case of disagreements, they are resolved through discussion or soliciting third-party opinions. Then, the Cochrane Systematic Reviewer's Handbook 5.0.1 was used to evaluate the methodological quality of the included studies using the bias risk assessment tool for RCTs.

### 2.5 Quality evaluation

According to the bias risk assessment method recommended by the Cochrane Collaboration, two researchers independently evaluate and discuss with each other to resolve any disagreements. The risk assessment of bias mainly includes: ① the generation of random allocation schemes; ② Allocation hidden; ③ Blinding the subjects and implementers; ④ Implement blinding for the evaluators of the results; ⑤ Result data integrity; ⑥ Selective Results Report; ⑦ Other biases arise. The evaluation results of each item are presented in terms of “low risk of bias”, “unclear risk of bias”, and “high risk of bias”, and are presented in a bias risk summary chart and a bias risk ratio chart.

### 2.6 Statistical analysis

Meta-analysis was conducted using RevMan 5.3 software provided by the Cochrane Collaboration. Categorical variables are analyzed using odds ratio (OR) or relative risk (RR), while quantitative data are analyzed using mean difference (MD) or standardized mean difference (SMD) as effect analysis statistics, with a 95% confidence interval (CI) provided. The heterogeneity among the included research results was analyzed using a chi square test. If there is statistical homogeneity (*P* > 0.1, I^2^ < 50%) among the research results, a fixed effects model will be used for meta-analysis. If there is statistical heterogeneity (*P* ≤ 0.1, I^2^ ≥ 50%) among the research results, a random effects model will be used for meta-analysis. If the heterogeneity is too large, only descriptive analysis will be conducted. Use STATA12.0 software for Egger linear regression to determine if there is publication bias.

## 3 Results

### 3.1 Basic information of literature

**Figure 1:**
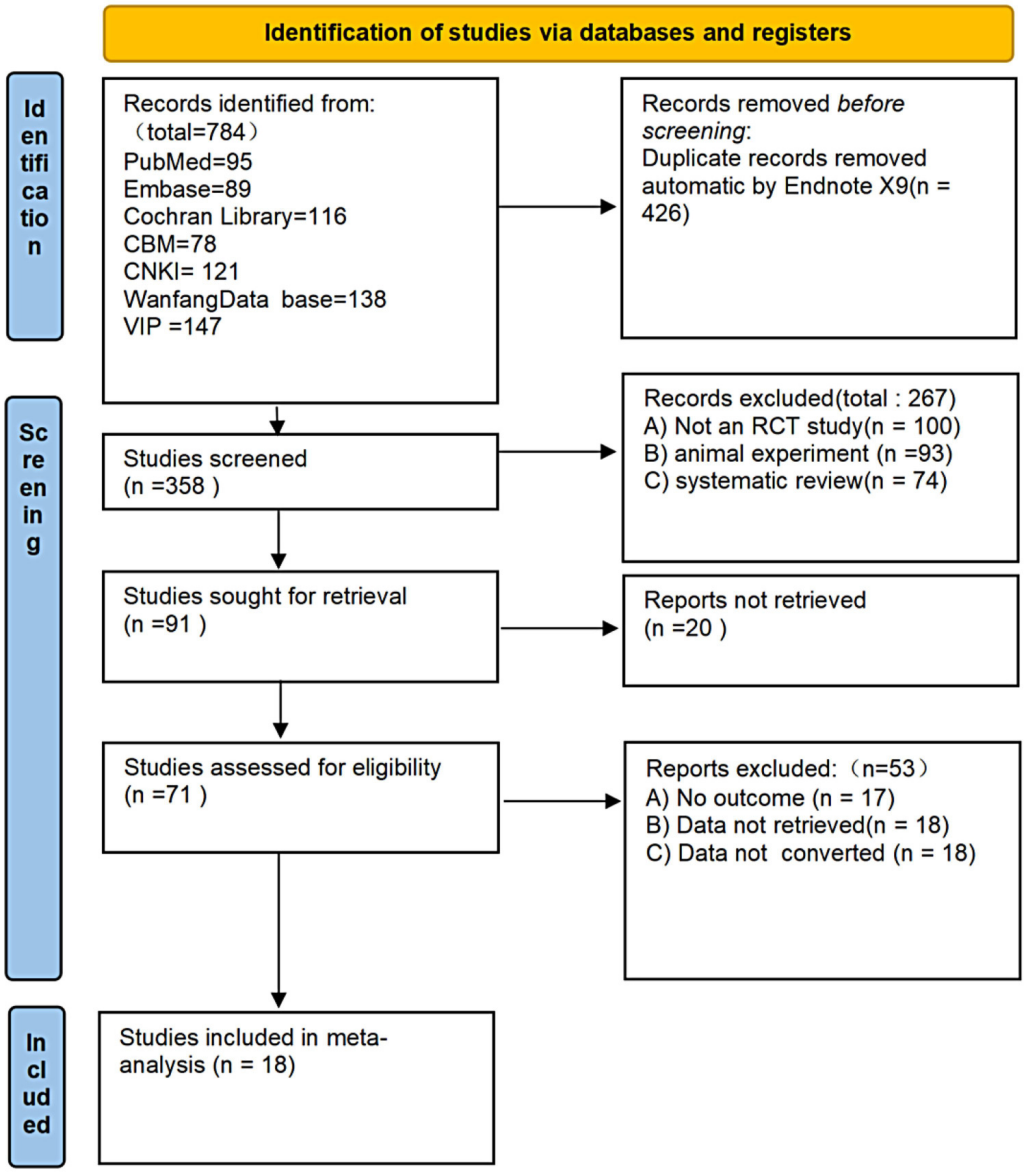
Literature screening process diagram.

A total of 784 articles were retrieved, and 426 duplicate articles were excluded, resulting in a preliminary total of 358 articles. 267 unrelated articles were excluded from the reading title and abstract, and then screened according to inclusion and exclusion criteria. Finally, 18 articles were included, totaling 1,326 patients (25, 27–43), as shown in Figure 1. The basic information of the included data is shown in Table 1.

**Table 1:** Basic information of included studies .

Author	Year	Grade of KOA	Age		Gender		Number of cases		Main observation indicators	Follow-up time
			PRP	HA	PRP	HA	PRP	HA		
AHMAD (28)	2018	Grade 1–3 (KL)	56.2 ± 6.8	56.8 ± 74	14/31	14/30	45	44	VAS, IKDC	6
Buendía (29)	2018	Grade 1–2 (KL)	56.15 ± 3.001	24.9 ± 0.32	16/17	15/17	33	32	WOMAC, VAS	12
Cerza (30)	2012	Grade 1–3 (KL)	66.5 (11.3)	66.2 (10.6)	25/35	28/32	60	60	WOMAC	6
Cole (31)	2016	Grade 1–3 (KL)	55.9 ± 10.4	56.86 ± 105	28/21	20/30	49	50	VAS, IKDC, WOMAC	6
Filardo (25)	2015	Grade 1–3 (KL)	53.32 ± 13.2	57.55 ± 11.8	60/34	52/37	94	89	EQ-VAS, Tgenerator	12
Gormel (34)	2015	Grade 1–3 (KL)	53.7 ± 13.1	28.7 ± 48	16/23	17/22	39	39	IKDC, EQ-VAS, rateSatisfaction	6
Huang (35)	2019	Grade 1–2 (KL)	54.5 ± 12	54.8 ± 11	25/15	19/21	40	40	VAS, WOMAC	12
Kon (36)	2011	Grade 1–4 (KL)	562 ± 6.8	54.9 ± 12.6	30/20	25/25	50	50	IKDC, EQ-VAS, rateSatisfaction	6
Lin (37)	2019	Grade 1–3 (Ahlback)	61.17 (13.08)	23.98 (2.62)	9/22	10/19	31	29	KDC, WOMAC	12
Louis (38)	2018	Grade2–3 (KL)	53.2 ± 11.7	48.5 ± 11.5	14/10	11/13	24	24	WOMAC, Satisfaction rate	6
Martino (32)	2012	Grade 1–3 (KL)	55	27	37/17	31/24	54	55	EQ-VAS, TgeneratIKDC	12
Matteo (25)	2015	Grade 1–3 (KL)	52.7 ± 13.2	27.2 ± 7.6	53/32	47/35	85	82	EQ-VAS, Tgenerator	24
Raeissadat (39)	2017	Grade2–3 (KL)	57.0 ± 7.18	59.5 ± 7.54	7/29	6/27	36	33	WOMAC, Satisfaction rate	6
Spakova (40)	2012	Grade 1–3 (KL)	52.80 ± 12.43	N/A	33/27	31/29	60	60	WOMAC	6
Su (41)	2018	Grade2–3 (KL)	54.16 ± 6.56 (37–71)	28.17 ± 143	11/14	12/18	25	30	WOMAC, VAS	18
Tavassoli (27)	2019	Grade 1–2 (KL)	66.04 ± 7.58	63.30 ± 8.87	6/22	9/19	28	27	WOMAC	6
Yaradilmis (42)	2020	Grade 1–3 (KL)	58.93 ± 6.25	32.53 ± 6.25	3/27	4/26	30	30	WOMAC, VAS	12
Yu (43)	2018	N/A	46.2 ± 8.6	51.5 ± 9.3	50/54	48/40	104	88	WOMAC	12

### 3.2 Results of bias risk assessment

**Figure 2:**
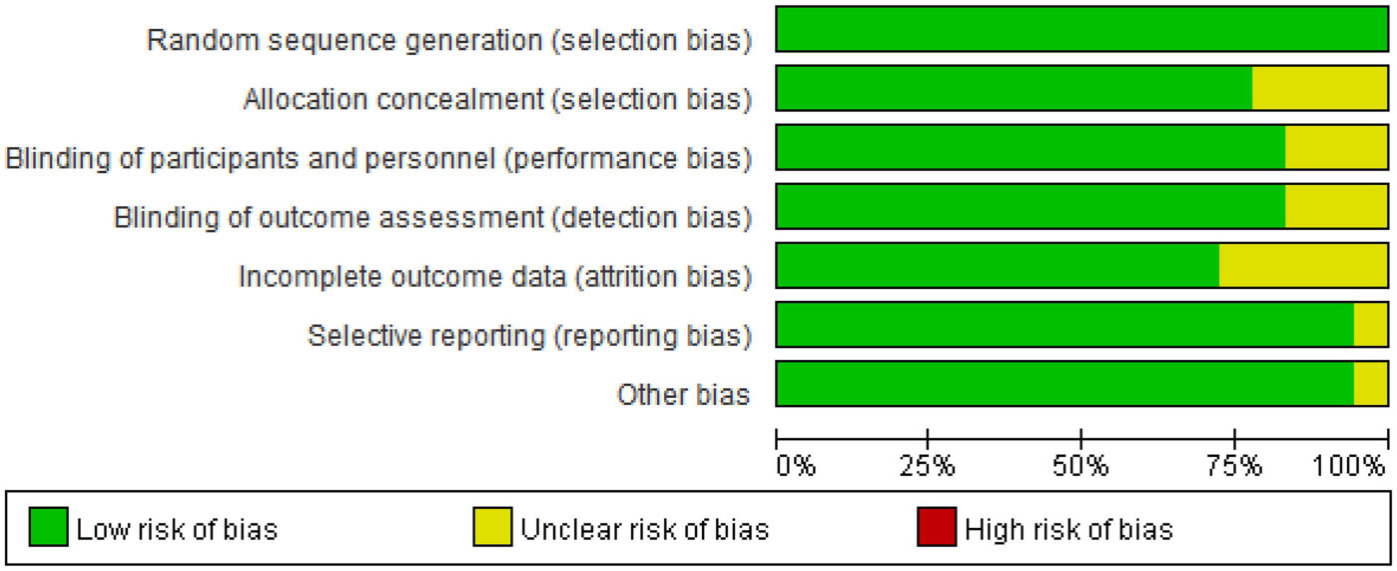
Risk of bias graph.

**Figure 3:**
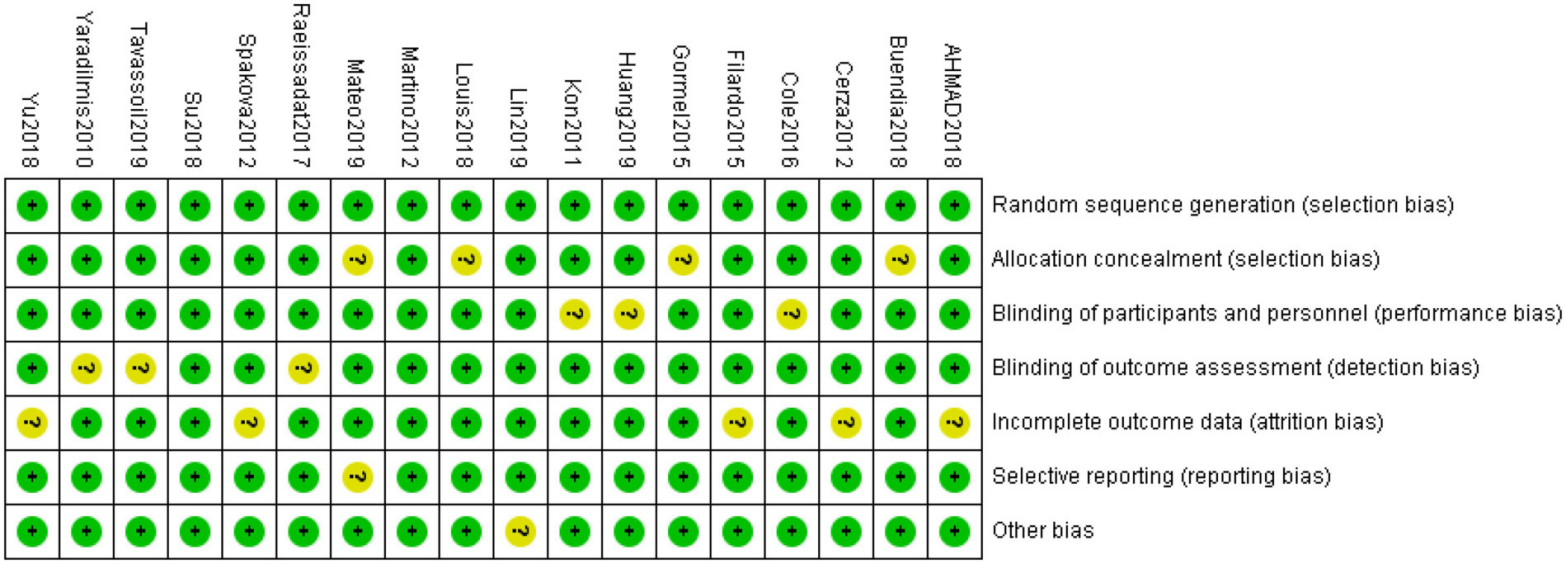
Risk of bias summary.

The 18 included literature were all randomized controlled trials (RCTs), with no high-risk bias literature and high literature quality. The specific bias risk assessment is shown in Figures 2, 3.

### 3.3 Meta-analysis results

#### 3.3.1 WOMAC rating

At one month after treatment, WOMAC was included in three studies, and heterogeneity tests were conducted on the literature. The results showed that the heterogeneity between the included studies was relatively small (*P* *=* 0.36, I^2^ = 3%). Using a fixed effects model with combined effect sizes, the meta-analysis results of the fixed effects model showed that the PRP group was superior to the HA group in WOMAC scores [*SMD = *−0.18, 95% *CI (*−0.43, 0.08), *P* *=* 0.18], but the difference was not statistically significant.

At 6 months after treatment, WOMAC was included in a total of 6 studies. Heterogeneity tests were conducted on the literature, and it was found that there was significant heterogeneity among the included studies (*P* *<* 0.0001, I^2^ = 97%). Using a random effects model with combined effect sizes, the meta-analysis results of the random effects model showed that the PRP group had better WOMAC scores than the HA group [*SMD = *−8.32, 95% *CI (*−11.43, −5.20), *P* *<* 0.0001], and the difference was statistically significant.

**Figure 4:**
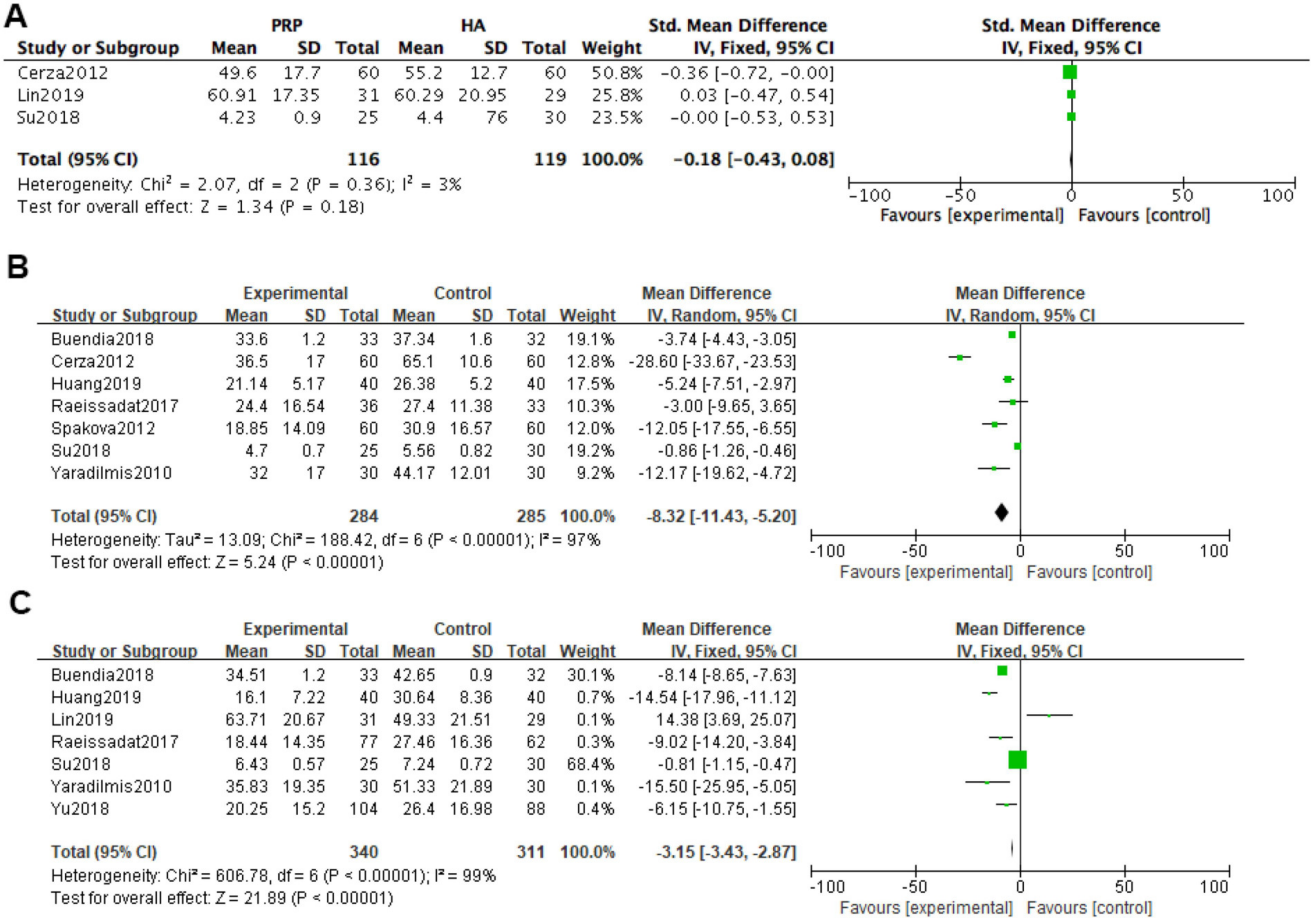
Meta-analysis forest plot of WOMAC score. **(A)** Treatment for one month. **(B)** Treatment for 6 months. **(C)** Treatment for 12 months.

At 12 months after treatment, WOMAC was included in a total of 7 studies, and heterogeneity tests were conducted on the literature. The results showed that there was significant heterogeneity among the included studies (*P* *<* 0.0001, I^2^ = 99%). Using a random effects model with combined effect sizes, the random effects model meta-analysis showed that the PRP group had better WOMAC scores than the HA group [*SMD = *−3.15, 95% *CI (*−3.43, −2.87), *P* *<* 0.0001], and the difference was statistically significant (Figure 4).

#### 3.3.2 IKDC rating

At 2 months after treatment, a total of 6 studies were included in the IKDC score. Heterogeneity tests were conducted on the literature, and it was found that there was significant heterogeneity among the included studies (*P* *=* 0.02, I^2^ = 63%). Using a random effects model with combined effect sizes, the meta-analysis results of the random effects model showed that the experimental group had better scores than the control group [*SMD = *−0.10, 95% *CI (*−0.35, 0.14), *P* *=* 0.40], but the difference was not statistically significant.

At 6 months after treatment, a total of 7 studies were included in the IKDC score. Heterogeneity tests were conducted on the literature, and it was found that there was significant heterogeneity among the included studies (*P* *<* 0.00001, I^2^ = 93%). Using a random effects model with combined effect sizes, the meta-analysis results of the random effects model showed that the experimental group was superior to the control group in terms of scoring [*SMD=*0.85, 95% *CI (*0.26, 1.43), *P* *=* 0.004], and the difference was statistically significant.

**Figure 5:**
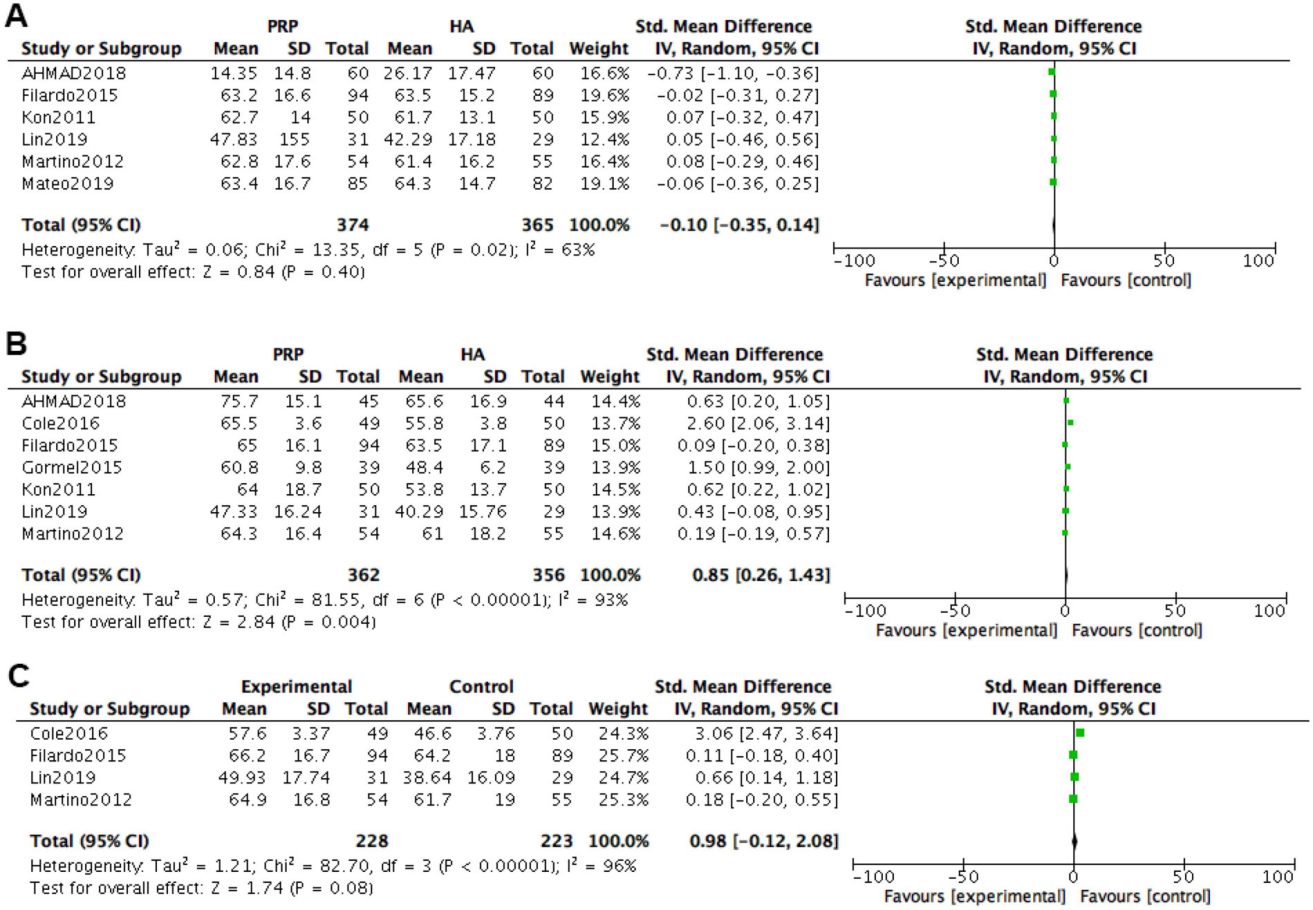
Meta-analysis forest plot of IKDC rating. **(A)** Treatment for 2 months. **(B)** Treatment for 6 months. **(C)** Treatment for 12 months.

At 12 months after treatment, a total of 4 studies were included in the IKDC score. Heterogeneity tests were conducted on the literature, and it was found that there was significant heterogeneity among the included studies (*P* *=* 0.0001, I^2^ = 96%). Using a random effects model with combined effect sizes, the meta-analysis results of the random effects model showed that the experimental group was superior to the control group in terms of evaluation scores [*SMD = *0.98, 95% *CI (*−0.12, 2.08), *P* *=* 0.08], but the difference was not statistically significant (Figure 5).

#### 3.3.3 VAS pain score

At 3 months after treatment, VAS pain scores were included in 3 studies. Heterogeneity tests were conducted on the literature, and it was found that the heterogeneity between the included studies was relatively small (*P* *=* 0.65, I^2^ = 0%). Using a fixed effects model with combined effect sizes, the meta-analysis results of the fixed effects model showed that the VAS scores of the PRP group were better than those of the HA group [*SMD = *−0.56, 95% *CI (*−0.84, −0.28), *P* *<* 0.0001], and the difference was statistically significant.

At 6 months after treatment, VAS pain scores were included in 4 studies. Heterogeneity tests were conducted on the literature, and it was found that there was significant heterogeneity among the included studies (*P* *=* 0.10, I^2^ = 52%). Using a random effects model with combined effect sizes, the meta-analysis results of the random effects model showed that the VAS scores of the PRP group were better than those of the HA group [*SMD = *−0.85, 95% *CI (*−1.21, −0.48), *P* *<* 0.0001], and the difference was statistically significant.

**Figure 6:**
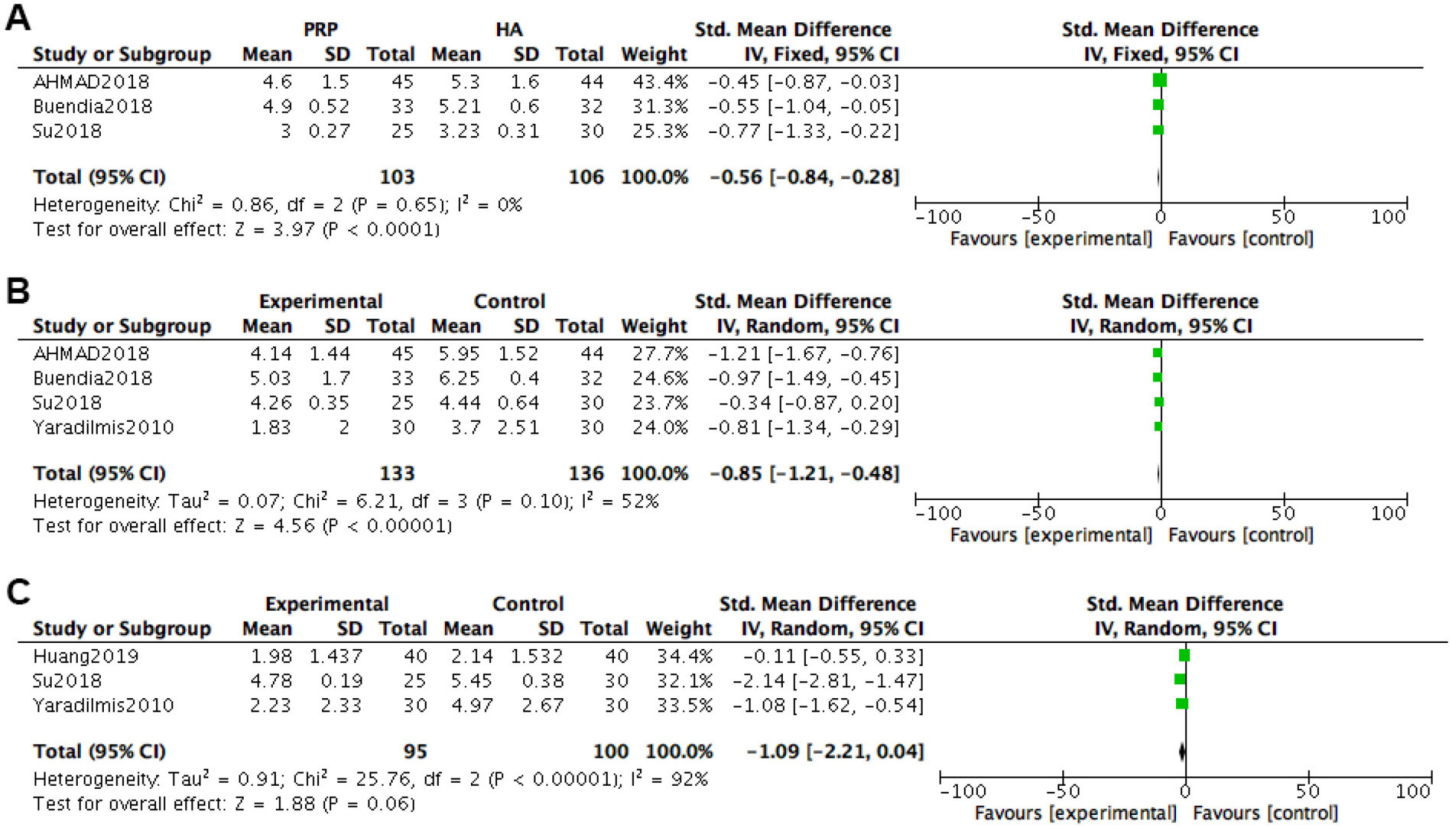
Forest plot of VAS scores for knee joint pain. **(A)** Treatment for 3 months. **(B)** Treatment for 6 months. **(C)** Treatment for 12 months.

At 12 months after treatment, VAS pain scores were included in three studies. Heterogeneity tests were conducted on the literature, and it was found that there was significant heterogeneity among the included studies (*P* *<* 0.00001, I^2^ = 92%). Using a random effects model with combined effect sizes, the meta-analysis results of the random effects model showed that the VAS scores of the PRP group were better than those of the HA group [*SMD = *−1.09, 95% *CI (*−2.21, 0.04), *P* *=* 0.06], and the difference was not statistically significant (Figure 6).

#### 3.3.4 WOMAC pain score

At 6 months after treatment, WOMAC pain score was included in 3 studies. Heterogeneity tests were conducted on the literature, and it was found that there was significant heterogeneity among the included studies (*P* *<* 0.00001, I^2^ = 93%). Using a random effects model with combined effect sizes, the meta-analysis results of the random effects model showed that the VAS score of the PRP group was better than that of the HA group [*SMD = *−1.67, 95% *CI (*−2.91, −0.43), *P* *=* 0.008], and the difference was statistically significant.

**Figure 7:**
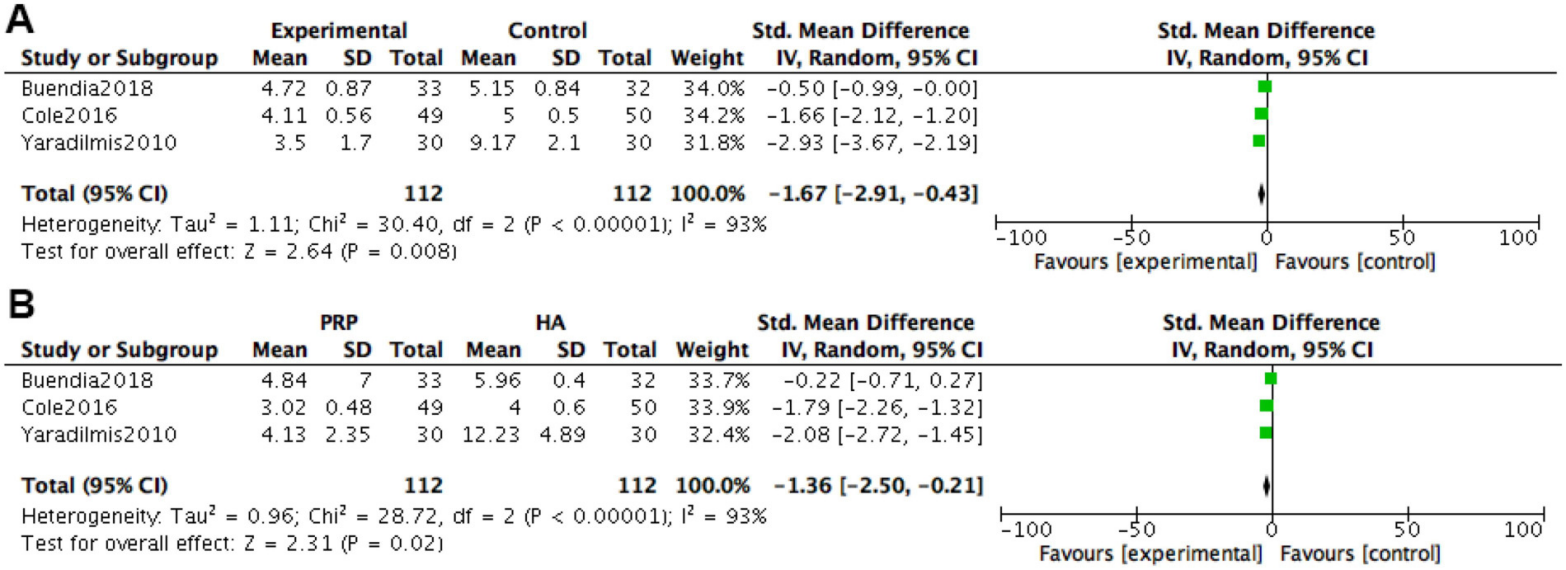
Forest plot of WOMAC score meta-analysis for knee joint pain. **(A)** Treatment for 6 months. **(B)** Treatment for 12 months.

At 12 months after treatment, WOMAC pain score was included in three studies. Heterogeneity tests were conducted on the literature, and it was found that there was significant heterogeneity among the included studies (*P* *<* 0.00001, I^2^ = 96%). Using a random effects model with combined effect sizes, the meta-analysis results of the random effects model showed that the VAS score of the PRP group was better than that of the HA group [*SMD = *−1.36, 95% *CI (*−2.50, −0.21), *P* *=* 0.02], and the difference was statistically significant (Figure 7).

#### 3.3.5 EQ-VAS rating

At 2 months after treatment, EQ-VAS pain scores were included in 3 studies. Heterogeneity tests were conducted on the literature, and it was found that there was no heterogeneity among the included studies (*P* *=* 0.81, I^2^ = 0%). The fixed effects model combined with effect size was used. The results of the fixed effects model meta-analysis showed that the VAS scores of the PRP group were better than those of the HA group [*SMD=*0.20, 95% *CI (*0.01, 0.38), *P* *=* 0.04], and the difference was statistically significant.

At 6 months after treatment, EQ-VAS pain scores were included in 4 studies. Heterogeneity tests were conducted on the literature, and it was found that there was no heterogeneity among the included studies (*P* *<* 0.00001, I^2^ = 98%). Using a random effects model with combined effect sizes, the meta-analysis results of the random effects model showed that the VAS scores of the PRP group were better than those of the HA group [*SMD = *−0.68, 95% *CI (*−1.99, 0.63), *P* *=* 0.31], and the difference was not statistically significant.

**Figure 8:**
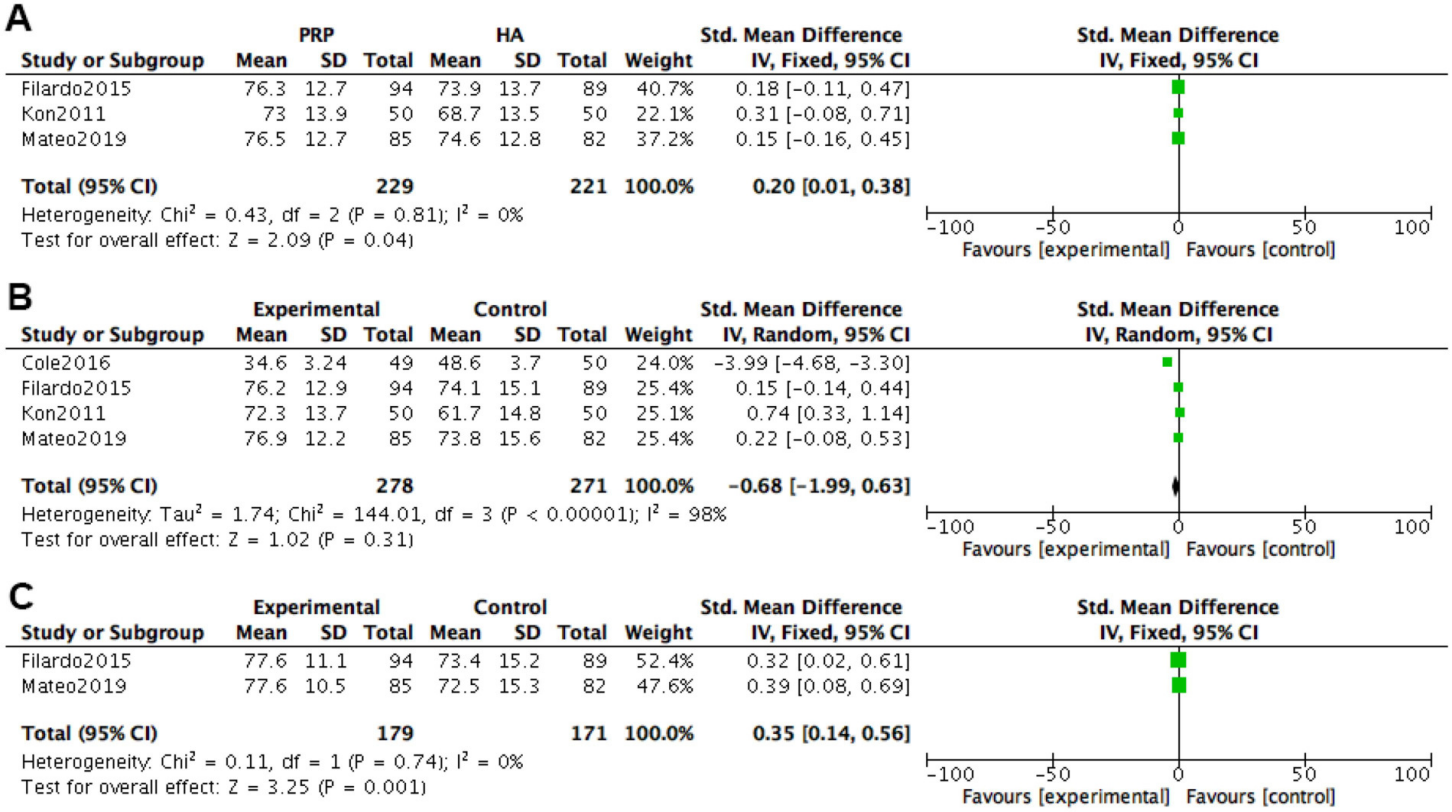
Meta-analysis forest plot of EQ-VAS score. **(A)** Treatment for 2 months. **(B)** Treatment for 6 months. **(C)** Treatment for 12 months.

At 12 months after treatment, EQ-VAS pain scores were included in two studies. Heterogeneity tests were conducted on the literature, and it was found that there was no heterogeneity between the included studies (*P* *=* 0.74, I^2^ = 0%). Using a fixed effects model with combined effect sizes, the meta-analysis results of the fixed effects model showed that the VAS scores of the PRP group were better than those of the HA group [*SMD = *0.35, 95% *CI (*0.14, 0.56), *P* *=* 0.001], and the difference was statistically significant (Figure 8).

#### 3.3.6 Knee joint movement score

At 2 months after treatment, knee joint motion scores were included in 2 studies. Heterogeneity tests were conducted on the literature, and it was found that there was no heterogeneity between the included studies (*P* *=* 0.97, I^2^ = 0%). Using a fixed effects model with combined effect sizes, the meta-analysis results of the fixed effects model showed that the VAS scores of the PRP group were better than those of the HA group [*SMD=*1.31, 95% *CI (*−0.01, 0.41), *P* *=* 0.06], and the difference was not statistically significant.

**Figure 9:**
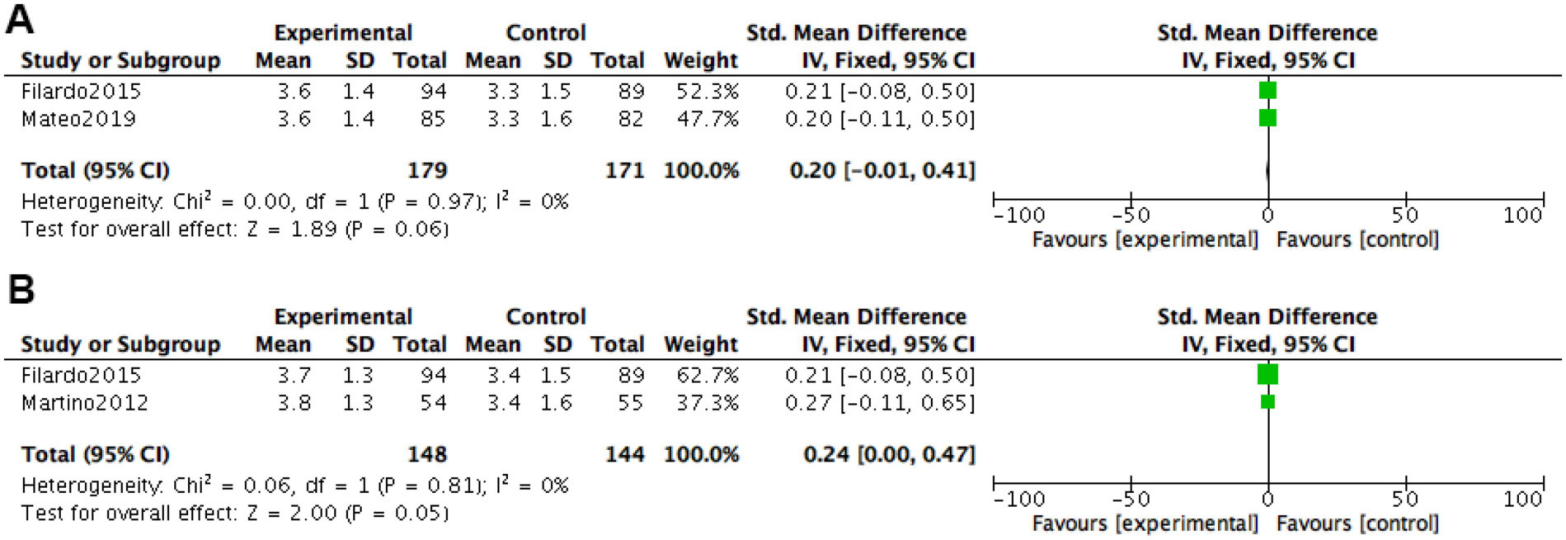
Meta-analysis forest chart of knee joint movement score. **(A)** Treatment for 2 months. **(B)** Treatment for 12 months.

At 12 months after treatment, knee joint motion scores were included in two studies. Heterogeneity tests were conducted on the literature, and it was found that there was no heterogeneity between the included studies (*P* *=* 0.81, I^2^ = 0%). The fixed effects model combined with effect size was used. The results of the fixed effects model meta-analysis showed that the VAS score of the PRP group was better than that of the HA group [*SMD=*0.24, 95% *CI (*0.00, 0.47), *P* *=* 0.05], and the difference was not statistically significant (Figure 9).

#### 3.3.7 Adverse reactions

**Figure 10:**
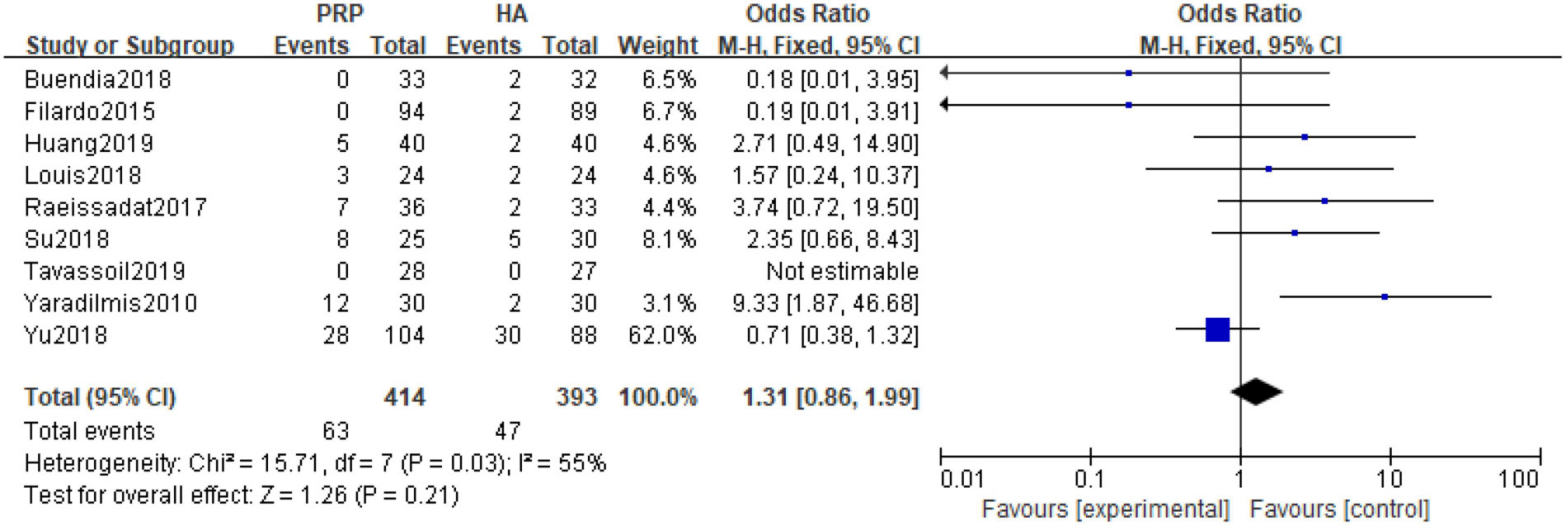
Meta-analysis forest plot of adverse reactions.

5 studies reported adverse reactions to injection of PRP and HA. Heterogeneity tests were conducted on the literature, indicating moderate heterogeneity (*P* *=* 0.03, I^2^ = 55%) in the included studies. Random effects models were used to combine effect sizes. Meta-analysis of the random effects model showed that the difference between PRP and HA was not statistically significant [*SMD=*1.31, 95% *CI (*0.86, 1.99), *P* *=* 0.21] (Figure 10).

#### 3.3.8 Subject satisfaction

**Figure 11:**
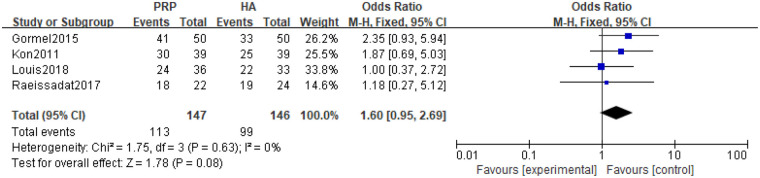
Meta-analysis forest chart of subject satisfaction.

5 studies reported on subject satisfaction and conducted heterogeneity tests on the literature, indicating moderate heterogeneity (*P* *=* 0.63, I^2^ = 0%) in the included studies. Using a random effects model with combined effect sizes, the meta-analysis results of the random effects model showed that there was no statistically significant difference between PRP and HA [(MD 1.60 [0.95, 2.69], *P* *=* 0.08] (Figure 11).

### 3.4 Analysis of publication bias

Due to the inclusion of the WOMAC score in the literature at 6 months of treatment, STATA12.0 software was used for Egger linear regression to determine whether there was publication bias. The Egger linear regression results showed that the test statistic *t* *=* −1.46, *P* *=* 0.182 > 0.05, indicating that there may not be publication bias, as shown in Table 2.

**Table 2:** Egger's method test calculation results.

Std_Eff	Coef.	Std. Err.	t	P>|t|	[95% Conf, Interval]	
Slope	5.025914	1.293667	3.89	0.005	2.042712	8.009115
Bias	−2.681423	1.83497	−1.46	0.182	−6.912871	1.550026

## 4 Discussion

The etiology of knee osteoarthritis is complex, characterized by joint friction sounds, degeneration of knee cartilage, and joint swelling. As the condition progresses, the patient's range of joint movement gradually becomes limited, which can affect daily life and make walking, climbing stairs, and other daily activities difficult. In more severe cases, joint deformation can occur, leading to gait abnormalities. Sodium hyaluronate injection into the knee joint cavity is commonly used in clinical practice to treat knee osteoarthritis. It can alleviate joint pain and improve joint function in the short term, but cannot prevent disease progression. Therefore, it is necessary to find a more comprehensive treatment plan to improve the treatment effect of knee osteoarthritis.

This study found that the efficacy of PRP is time-dependent, which is highly consistent with the conclusions of multiple large-scale meta-analyses and consensus guidelines in recent years.

Our results indicate that at the 6-month follow-up, PRP significantly outperformed HA in terms of WOMAC total score, pain score, and IKDC functional score. This finding aligns with the results of a meta-analysis published in 2025, which encompassed 42 RCTs and clearly demonstrated that PRP outperforms HA in terms of WOMAC total score and physical function score at both 6 and 12 months post-treatment (44). More importantly, the latest authoritative consensus from the European Society for Sports Traumatology, Knee Surgery, and Arthroscopy (ESSKA) is based on ample evidence and explicitly recommends the preferential use of PRP over HA in the treatment of KOA (recommendation level B), bolstering our conclusion from a clinical guideline perspective (45).

We found that the advantages of PRP in some indicators diminished at 12 months, which is related to the heterogeneity of research in this field. A recent meta-analysis provides a new perspective on long-term efficacy, indicating that at 1-year follow-up, combined injection of PRP and HA is most effective in relieving pain and improving function, surpassing any single therapy (46). This suggests that the focus of future research may shift from “which is better, PRP or HA” to “how to optimize the combined treatment regimen”, and also provides a reasonable explanation for our results—combined therapy may be the key to achieving sustained efficacy.

After binding to the transmembrane receptors outside the cartilage cell membrane, growth factors activate relevant signaling pathways and regulate the expression of intracellular genes, thus promoting the proliferation of chondrocytes, the formation of matrix and collagen synthesis (47). In addition, relevant studies have shown that PRP promotes chondrogenesis mainly by inducing the replication of target genes and the expression of type II collagen mRNA (45, 48). PRP is rich in growth factors including platelet-derived growth factor (PDGF), insulin-like growth factor (IGF), transforming growth factor—*β* (TGF- *β*), etc (49). These growth factors can recruit chondrogenic cells and stimulate cartilage proliferation, while promoting cartilage matrix synthesis (50). While HA mainly improves knee function by lubricating the knee joint, there is no evidence that it can reduce the inflammatory response. From the mechanism of action of both, it can be shown that PRP treatment of early knee osteoarthritis shows that it is mainly through changing the wear of articular cartilage and repairing the worn cartilage tissue (51). For the WOMAC score and IKDC score, the included studies are statistically heterogeneous. The reason for the heterogeneity may be that different researchers have subjective differences in the WOMAC score and IKDC score, and different researchers may have different scores. Therefore, PRP has obvious advantages and more stable and lasting effects than HA in the treatment of knee osteoarthritis.

According to the results of meta-analysis, there was a difference between PRP and HA in the improvement of knee pain (VAS pain score) at the 3rd and 6th month of follow-up. PRP could better alleviate joint pain than HA, but there was no significant difference between PRP and HA in the improvement of function (VAS pain score) at the 12th month. It was considered that PRP was not superior to HA in the improvement of long-term pain function; At the 6th and 12th month of follow-up, PRP and HA showed differences in pain improvement (WOMAC pain score), and PRP could better alleviate joint pain than HA; In the second, sixth, and twelfth months of follow-up, PRP and HA showed differences in health evaluation (EQ-VAS score), and PRP could better improve the health status of patients than HA. It shows that PRP has a significant effect on reducing the pain of knee osteoarthritis compared with HA. The concentration of inflammatory regulators interferon (IFN), such as (IL-4, IL-10, ifn- *γ*, etc.) and interleukin (IL), in PRP is directly related to the regulation of sterile inflammatory response in the joint (52). In addition, growth factors can inhibit the inflammatory response. Therefore, injecting PRP into the knee joint through the articular cavity can improve the soft tissue inflammation of the degenerated cartilage of the knee joint to a certain extent, promote the growth and proliferation of chondrocytes, and regulate the local microenvironment of the knee joint (53). It mainly regulates the microenvironment of the knee joint by repairing the connective tissue around the joint and changing the composition of the joint fluid. Relevant studies have shown that PRP can adjust the concentration of active proteins around the joint and reduce the proliferation of synovium in the joint. At the same time, it can also affect the excitability of tissues and inhibit the growth of bacteria (54). Although HA can relieve the pain and inflammation of the knee joint, it does not have the effect of growth factors to promote the proliferation of chondrocytes and reduce the degradation and destruction of articular cartilage. There is no such factor in HA, so PRP can repair osteoarthritis specifically (22).

We also found that compared to HA, PRP did not increase the risk of adverse events. The main adverse reactions of PRP treatment for knee osteoarthritis include knee pain, swelling, and functional limitation. Most reported adverse reactions last for several minutes to hours and resolve within a few days after treatment, which is consistent with the conclusions of most studies (55, 56). The main PRP-related adverse reactions are transient, self-limited mild to moderate pain, swelling, and functional limitation at the injection site, usually occurring within minutes to hours and resolving spontaneously within a few days. Serious adverse events are extremely rare in reported literature (57).

We conducted a more in-depth exploration of factors that may affect safety. The type of PRP is an important factor. Studies have shown that leucocyte-poor PRP (LP-PRP) may induce less post-injection pain than leucocyte-rich PRP (LR-PRP). In addition, the preparation process is also crucial (58). For example, PRP prepared by double centrifugation may retain fewer leucocytes and inflammatory mediators than that prepared by single centrifugation, thereby reducing adverse reactions (59). The clarification of these factors helps clinicians further enhance the safety of PRP treatment by optimizing techniques.

In addition to comparing PRP with HA, we place the safety of PRP in a broader context of conservative treatment. The ESSKA consensus clearly states that compared to corticosteroids (CS) commonly used clinically, PRP not only has a longer duration of efficacy but also lacks cartilage toxicity and has fewer complications, making it a safer option that can bring long-term clinical improvement (45). This comparison greatly highlights the safety advantages of PRP in long-term disease management.

This study not only statistically showed that PRP group was better than HA group, but also had clinically significant differences in WOMAC total score and IKDC score between the two groups. Previously, Angst et al. found that the maximum value of the minimum clinically important difference (MCID) in the WOMAC total score measurement was 6% (60). In addition, Irrgang et al. also pointed out in the study that the absolute change of MCID of IKDC score at 6 months was 6.3 points (61). According to this meta-analysis, our WOMAC total score results showed that the PrP group significantly exceeded the MCID value pointed out by angst et al. at 6 and 12 months. In addition, the IKDC score of the PRP group had a significant advantage in clinical practice. These two results show that the results of this study are not only statistically different, but also prove that our results are also clinically significant.

Traditional views suggest that PRP primarily promotes cartilage repair by releasing growth factors. Recent studies have further revealed its potent anti-inflammatory and immunomodulatory effects. A recent study indicates that PRP can promote the transformation of pro-inflammatory M1 macrophages into anti-inflammatory M2 macrophages by inhibiting the NF-*κ*B signaling pathway, thereby reducing the levels of pro-inflammatory factors in synovial tissue and increasing the expression of anti-inflammatory factors. This mechanism not only explains the significant effects of PRP in alleviating pain and swelling but also elevates its role from mere repair to regulating the joint microenvironment, which is fundamentally different from the mechanism of HA, which primarily provides lubrication (62).

In this study, the effectiveness of PRP treatment was further explored in order to better clinical application. This study carried out strict quality control in terms of methodology, including literature screening, data extraction, quality assessment and other links, and followed internationally recognized standards and norms. This ensures the accuracy and reliability of the research results, and also improves the novelty and scientificity of the research. This study also explored the mechanism of PRP and HA in the treatment of KOA, providing theoretical support for understanding the difference in the efficacy of these two treatments. This not only helps to promote the research progress in related fields, but also provides new ideas and methods for future clinical practice.

The literature included in this study has the following limitations: first, in addition, the literature included in this paper lacks multicenter, large sample randomized controlled trials, and the credibility of the results has certain limitations; Second, there are certain differences in PRP concentration, HA concentration and treatment cycle in the included literature, which may have an impact on the efficacy of treating KOA. Third, although this article is strictly screened, due to the technical differences of operators and different treatment cycles, it may lead to publication bias, which needs to be supplemented with more literatures for meta-analysis in the follow-up, so as to draw more accurate conclusions. Finally, due to the time required for database indexing and our analysis timeline, it is possible that the most recently published studies may not have been captured. Future updates should consider including studies published after October 2024.

In conclusion, PRP has a better curative effect in the treatment of KOA, which is conducive to the functional improvement and pain relief of patients with koa, and can effectively improve the quality of life of patients. Future research should focus on standardizing PRP preparation procedures, exploring combined strategies involving PRP and drugs such as HA, and identifying populations with superior responses, thereby optimizing clinical treatment decisions for KOA.
